# Effect of various nitrogen conditions on population growth, temporary cysts and cellular biochemical compositions of *Karenia mikimotoi*

**DOI:** 10.1371/journal.pone.0171996

**Published:** 2017-02-22

**Authors:** Yan Zhao, Xuexi Tang, Xiaowei Zhao, You Wang

**Affiliations:** Department of Marine Ecology, Ocean University of China, Qingdao, China; University of Connecticut, UNITED STATES

## Abstract

The harmful algal bloom (HAB)-forming dinoflagellate *Karenia mikimotoi* was exposed to different nitrogen (N) conditions, in order to study the population growth, temporary cyst production and cellular biochemical compositions in laboratory. The results indicated the population growth of *K*. *mikimotoi* was inhibited by different levels of N starvation but showed similar fast recovery after the resupplement of N, and temporary cysts were induced in the period of N starvation. *K*. *mikimotoi* grew well in inorganic (NO_3_^-^, NO_2_^-^ and NH_4_^+^) and organic (urea) nitrogen sources, but the growth parameters (*K*, *T*_*p*_, *r*) showed differences when simulated by Logistic model regressions. When the cellular organic compounds were measured simultaneously, *K*. *mikimotoi* cultured in urea produced more short-chained fatty acids while *K*. *mikimotoi* cultured in NH_4_^+^ produced more non-fatty acids compounds, indicating the potential change of toxins production cultured by various N sources. We concluded that *K*. *mikimotoi* could adapt to fluctuating N environments typical of coastal environments including total N concentration (deficiency or recovery) and relative compositions (different N sources).

## Introduction

In the past several decades, the increased frequency and distribution of harmful algal blooms (HABs) in marine ecosystems has attracted worldwide research attention [1, 2, 3]. The heightened occurrence of HABs is primarily caused by eutrophication, which closely links human activities such as increased energy demand of the human population, elevated usage of nitrogen (N) and phosphate (P) fertilizers and expanded aquaculture industries [4, 5]. As the most essential and frequent limiting nutrients, the concentration of N, P and N/P ratios will influence the population dynamics and the physiology of harmful algae at the species level [3, 6]. It is commonly accepted that N limitation (NO_3_^-^, NO_2_^-^, NH_4_^+^ and organic N (e.g., urea and amino acids)) occurs more frequently in marine ecosystems compared to freshwater ecosystems [7, 8, 9]. In coastal waters the increased concentration of the urea and reduced N forms is primarily due to human influence. Urea can account for over 50% of the total N input in various coastal areas, thus the role of multiple N forms on the proliferation of HABs becomes more important than a single N form [3, 10, 11, 12].

Dinoflagellates are specialized to adapt to unfavorable environments, by forming cysts, leading to diverse competitive abilities for these species [13, 14, 15]. Dinoflagellates are capable of forming both resting and temporary cysts, with resting cysts formed via sexual reproduction and temporary cysts from asexual reproduction. Both cyst types may be induced by physical disturbance, extreme temperature fluctuations, darkness, bacteria, nutrient stress etc. [15, 16, 17]. Compared to resting cysts, temporary cysts can quickly transition to vegetative cells when the ambient environments become favorable [14]. The encystment of dinoflagellates has been considered an important role in the maintenance of algal blooms during fluctuating environmental factors [18, 19, 20]. Marasović (1989) found temporary cysts of *Gonyaulax polyedra* Stein formed in Kaštela Bay (located at the eastern Adriatic coast) when environmental conditions changed dramatically, which was important for the persistence and recurrence of the blooms of this species. Garcés et al. (1998) reported that temporary cysts could play a pivotal role in the maintenance of massive blooms of *Alexandrium taylori* Balech in the north-west Mediterranean coast [21, 22].

*K*. *mikimotoi* is a common harmful algal species in worldwide marine ecosystems, especially in Asian areas [23, 24]. The occurrence of *K*. *mikimotoi* blooms have been reported along the west coast of Japan, east and south coasts of China, south Atlantic and east coast of USA and European coastse [1, 23, 25]. *K*. *mikimotoi* is evidenced to release hemolytic toxins and ichthyotoxins which threaten local fisheries and the health of the food web [26, 27]. Former studies indicated different nitrogen sources for HAB species could lead to the release of different toxins [28, 29]. As mentioned above, phytoplankton in coastal areas are not only experiencing enhanced eutrophication, but also changes in nutrient composition. The adaptation of *K*. *mikimotoi* to varying N conditions in relation to the occurrence of *K*. *mikimotoi* blooms, requires further investigation. In laboratory controlled conditions, we studied the response of *K*. *mikimotoi* to N starvation and recovery utilizing different forms of N. To determine how changing N conditions influence the *K*. *mikimotoi* bloom formation and toxin production, we analyzed population dynamics, formation of temporary cysts and fatty acid composition.

## Material and methods

Experimental microalgal species of *K*. *mikimotoi* was provided by the Algal Center of the Institute of Oceanography, Chinese Academy of Sciences. This strain of *K*. *mikimotoi* was originally isolated from East China Sea (ECS) and it is clonal. *K*. *mikimotoi* was cultured with pre-filtered natural seawater from coastal Qingdao (pH: 8.0±0.2; salinity: 30), enriched with f/2 nutrients, trace elements and vitamins and sterilized (30 minutes, 121°C) before innoculation [30]. The irradiance and temperature in the incubator were 75 μmol photons m^-2^ s^-1^ and 19±1°C (respectively), under a light: dark cycle of 12h: 12h. 250-mL Erlenmeyer flasks were used for culturing species and the population was maintained at exponential growth phase via semi-continuous culture mode before experiments.

### N starvation and recovery experiments

Six treatments were designed with different initial nitrogen (NO_3_^-^) concentrations for the N starvation experiment (Table 1). All the treatments were performed in triplicate. The initial cell concentrations for *K*. *mikimotoi* were 1×10^4^ cells mL^-1^, and the culturing period to the stationary phase was around 20 days. The recovery experiment began at the termination of the starvation experiment. At the end of the N starvations experiment cultures were taken out and diluted to 1×10^4^ cells mL^-1^ with nutrient replete f/2 medium. Cell densities were counted daily and cellular chl *a* concentration were measured at different time points during both the starvation and recovery experiments.

**Table 1:** Different N: P rations (mol: mol) in N starvation experiments.

Treatment	NO_3_^-^ (μmol L^-1^)	PO_4_^3-^ (μmol L^-1^)
**0**	0	36
**N:P = 1:1**	55	36
**N:P = 2:1**	110	36
**N:P = 4:1**	220	36
**N:P = 8:1**	440	36
**control**	880	36

Microalgal cells were counted using a hemocytometer under a microscope OLYMPUS CX31, shape and size were observed and recorded using an inverted microscope (IX51 Olympus, Japan) to monitor the formation of temporary cysts. For spectrophotometric chl *a* measurements, 20 mL cultures were filtered onto GF/F glass fiber filters (Whatman) and pigments were extracted with 90% cold acetone overnight in the dark at -20°C [31].

### Varying N source experiments

For this experiment, we applied four different forms of N sources, including NaNO_3_, NaNO_2_, NH_4_Cl and (NH_2_)_2_CO (urea). The N, P, trace metals and vitamins concentrations were set to that of f/20 media (N: 88.23μmol L^-1^, P: 3.6μmol L^-1^) [32]. All treatments were performed in triplicate and cultured to the stationary phase (around 20 days). The initial cell density and the culture volume were set to 1.0×10^4^ cells mL^-1^ and 250 mL, respectively. Cell numbers were counted daily using a hemocytometer.

At the end of the experiment cells were collected to measure cellular fatty acids (FA) compositions. For FA analysis, 150–200 mL of culture was centrifuged to form pellets. The pellets were collected and stored in -20°C until Gas Chromatography—Mass Spectrometer (GC-MS) analysis. The cell pellets were broken by ultra—sonication (33kHz, 300W, 30 mins) in a solvent of ethyl acetate and petroleum ether (v: v = 1:1) to extract the FAs. This procedure was repeated three times and the extract was transferred to thick-walled glass tubes with Teflon-lined screw caps. Samples were then concentrated by centrifugation (4500 rpm, 15 mins). The supernatant was gently removed and the remaining liquid was evaporated under a stream of nitrogen gas. The resulting pellets containing algal FAs were converted to FA methyl esters (FAMEs) with the addition of 1 mL KOH-CH_3_OH (0.5 mol L^-1^), sealed with Teflon-lined screw caps under a stream of nitrogen gas and saponified for 15 mins at 70°C. After cooling, 1 mL boron trifluoride was added to each sample, followed by sealing, shaking and methanolysis for 30 mins at 70°C. 2.0 mL hexane and 2.0 mL saturated sodium chloride were added to each sample after the additional cooling step. The supernatant was collected and evaporated with nitrogen gas. The resulting 1 mL samples were stored at -80°C until GC-MS was conducted with an Agilent 5975C-7890A GC-MS instrument.

### Statistics

All the statistical analyses were performed with SPSS16.0 and figures were plotted by Sigmaplot 12.3. The means and standard errors (S.E.) in the figures and tables were calculated from the triplicates for each treatment. Growth curves were regressed by the Logistic growth model [33] (Eq 1). Temporary cysts production rate (*PC*) and cell inhibition rate (*PD*) under N starvation were calculated according to the method of Fistarol et al. [34] from Eqs 2–5.

One-way analysis of variance (one-way ANOVA) was used to determine significance among treatments, in which LSD test was used to group-paired significance after the test of variance homogeneity.

$$N_{t}=\frac{K}{1+e^{a-rt}},$$ Equation 1

where *N*_*t*_ is the cell density at time *t* (×10^4^ cells·mL^-1^); *K* is the carrying capacity of the population (×10^4^ cells·mL^-1^); *t* is the time (day); *a* is a constant equalling to *ln (K-N*_*0*_*)/ln (N*_*0*_*)*;*N*_*0*_ is the initial cell density of the population (×10^4^ cells·mL^-1^); *r* is the intrinsic growth rate or the maximum specific growth rate (day^-1^).

NIcont−Nnde=TA, Equation 2

where *NIcont* is the intact cell number in the control; *Nnde* is the intact cell number in the treated group; *TA* is the affected cell number because of the environmental nutrient changes.

TA−C=D, Equation 3

where *C* is the number of temporary cysts; *D* is the decreased amount of cell number due to N deficiency.

$$PD=\frac{D\times 100}{NIcont},$$ Equation 4

where *PD* is the cell inhibition rate.

$$PC=\frac{C\times 100}{NIcont},$$ Equation 5

where *PC* is percentage of cysts production.

## Results

### Population growth when exposed to N starvation and recovery conditions

**Fig 1:**
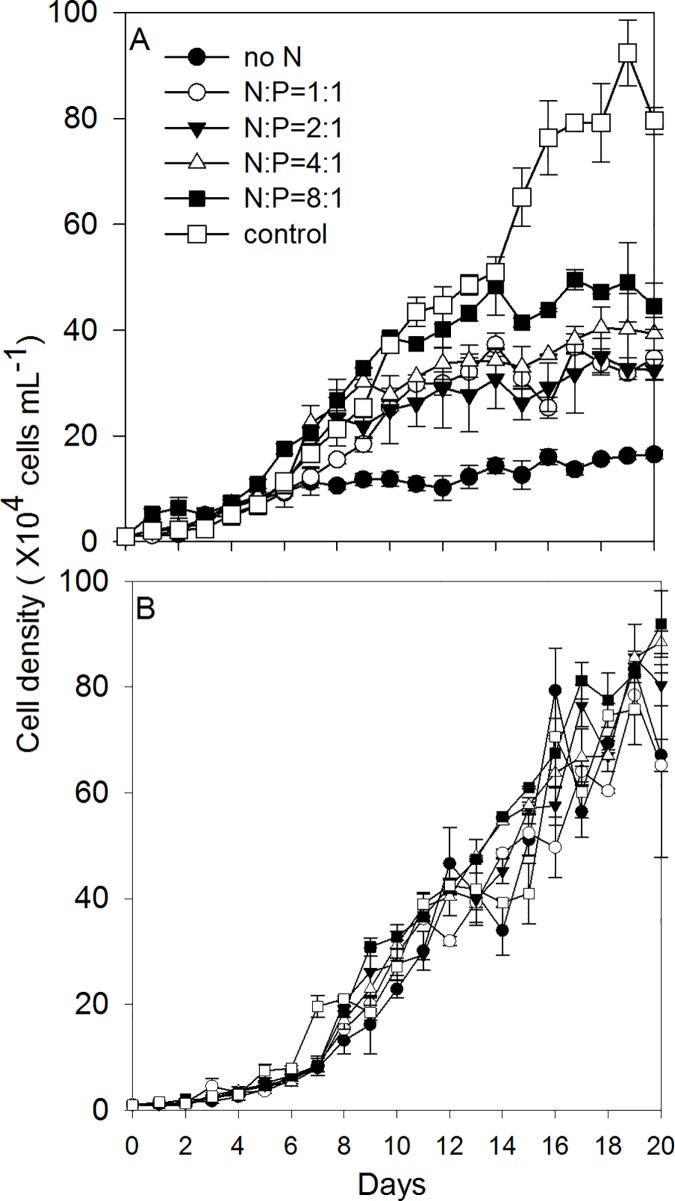
The population growth of *K*. *mikimotoi* under the N starvation condition (a) and the recovery condition (b) in different N: P ratios.

The population growth of *K*. *mikimotoi* under different N/P ratios and the recovery growth after the resupplement of N are shown in Fig 1. It took 20 days for the population to reach the stationary phase in both experiments. The population growth was significantly inhibited by N deficiency with the initial N concentration ranged from 0 μmol L^-1^ to 440 μmol L^-1^. The lower initial N concentration exhibited more inhibited population growth (Fig 1A). We calculated the regressed parameters by the Logistic model to quantify the growth dynamics in different treatments (Table 2). Based on the calculated Logistic parameters, the carrying capacity (*K*) values in all N deficient treatments were significantly lower (14.8 cells mL^-1^) than the control group (94.5 cells mL^-1^) (one-way ANOVA, F = 5.923, *P* = 0.006) (Table 2). The inflection time point (*T*_*p*_; where population growth rate started to decline), showed a decrease in the lower initial N concentration treatments (day 5.7-day 7.3) compared to the control treatment (day 12.2) (Table 2). The maximum specific growth rate (*r*) did not vary significantly between treatments (one-way ANOVA, F = 1.933, *P* = 0.162) (Table 2).

**Table 2:** The regressions of the Logistic model on *K*. *mikimotoi* population growth when exposed to N starvation conditions.

Population growth parameters	Growth equation	Carrying capacity *K* (×10^4^ cells mL^-1^)	*r* (d^-1^)	*T*_*p*_ (d)	R^2^
**No N**	*N* = 14.82/(1+e^(3.364–0.591*t*)^)	14.82*	0.591	5.7	0.914
**N:P = 1:1**	*N* = 31.36/(1+e^(3.623–0.577*t*)^)	31.36*	0.578	6.2	0.975
**N:P = 2:1**	*N* = 33.89/(1+e^(3.577–0.515*t*)^)	33.89*	0.515	6.9	0.961
**N:P = 4:1**	*N* = 37.33/(1+e^(3.533–0.498*t*)^)	37.33*	0.498	7.0	0.975
**N:P = 8:1**	*N* = 47.05/(1+e^3.263–0.444*t*)^)	47.05*	0.444	7.3	0.985
**N:P = 16:1**	*N* = 94.51(1+e^(3.767–0.308*t*)^)	94.51	0.308	12.2	0.984

^*^ indicated there was a statically significant difference between this treatment and the corresponding control group (*P*<0.05).

After the replenishment of N, cells in different treatments showed similar recovery growth patterns (Fig 1B). Although the cells were under different levels of N starvation, the calculated Logistic parameters (*K*, *r* and *T*_*p*_) after the N replenishment, were similar across the six treatments (Table 3). Compared to the parameters in the N starvation phase, *K* values in N = 0, N:P = 1:1, N:P = 2:1, N:P = 4:1 and N:P = 8:1 treatments increased 442.87%,171.23%, 184.64%, 144.82% and 94.25% in the recovery phase, respectively.

**Table 3:** The regressions of the Logistic model on *K*. *mikimotoi* population growth when exposed to N recovery conditions.

Population growth parameters	Growth equation	Carrying capacity *K* (×10^4^ cells mL^-1^)	*r* (d^-1^)	*T*_*p*_ (d)	R^2^
**No N**	*N* = 80.43/(1+e^(4.469–0.352*t*)^)	80.43	0.352	12.7	0.943
**N:P = 1:1**	*N* = 85.06/(1+e^(3.811–0.285*t*)^)	85.06	0.285	13.4	0.971
**N:P = 2:1**	*N* = 96.47/(1+e^(3.898–0.283*t*)^)	96.47	0.283	13.8	0.982
**N:P = 4:1**	*N* = 91.40/(1+e^(3.987–0.310*t*)^)	91.40	0.310	12.9	0.986
**N:P = 8:1**	*N* = 91.40/(1+e^(3.982–0.309*t*)^)	91.40	0.309	12.9	0.969
**N:P = 16:1**	*N* = 94.51/(1+e^(3.581–0.261*t*)^)	94.51	0.261	13.7	0.998

**Fig 2:**
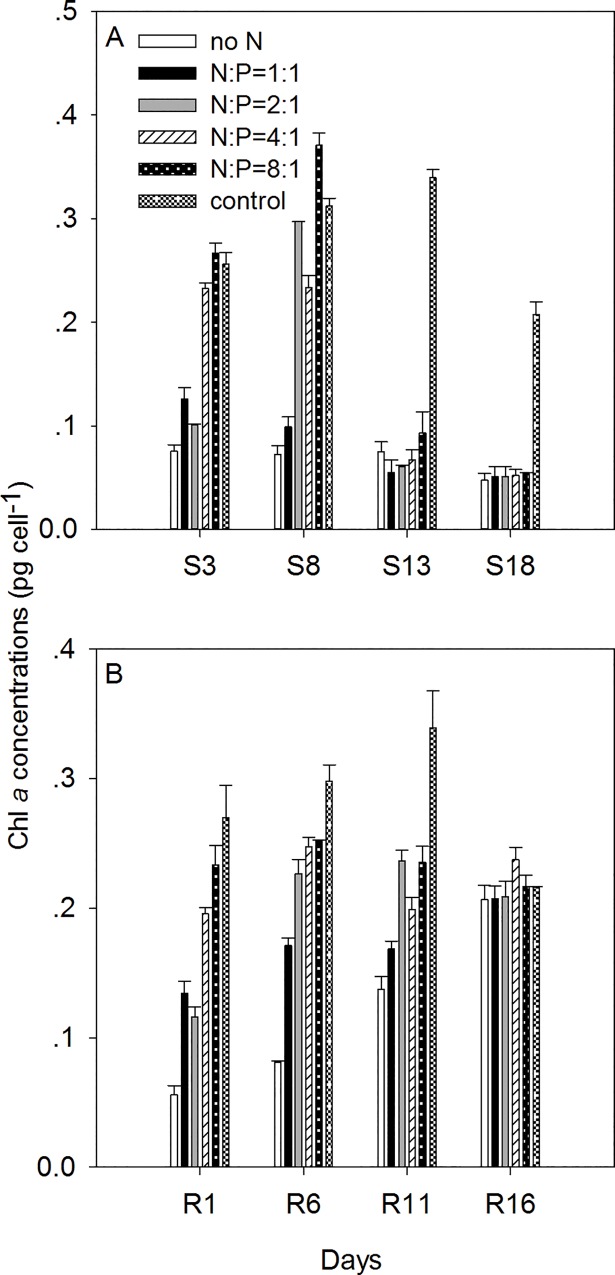
The changes of cellular chl *a* concentrations in the N starvation phase and the recovery phase under different N/P ratios. S3-S18 indicated the N starvation phase while R1-R16 indicated the recovery phase.

The chl *a* concentrations of individual cells were measured under the N starvation phase (S1-S18) and the recovery phase (R1-R16) (Fig 2). Chl *a* concentrations of *K*. *mikimotoi* cells were 0.28±0.05 pg cell^-1^ under normal conditions. In the N starvation phase, chl *a* concentrations started to decline on day S3 in the treatments N = 0, N:P = 1:1, N:P = 2:1 and N:P = 4:1 (*P*<0.001 for N = 0, N:P = 1:1, N:P = 2:1 treatments and *P* = 0.001 for N:P = 4:1 treatment, Fig 2A), while there was no significant change of cellular chl *a* content in the N:P = 8:1 treatments on day S3 (*P* = 0.078, Fig 2A). At the end of the experiment (day S18), the cellular chl *a* concentrations showed similar values in all N starvation treatments, which averaged 74.5% less than the control (*P*<0.01). In the recovery phase, all treatments recovered to values similar to the control after 16 days. After the replenishment of N, cellular chl *a* in the treatments of N:P = 1:1, N:P = 2:1, N:P = 4:1 and N:P = 8:1 showed an increase within 1 day (day R1), while chl *a* contents in the N = 0 treatment did not start to increase until day R6. On day R16, cellular chl *a* content in all treatments recovered to values similar to the control group (one-way ANOVA, F = 1.097, *P* = 0.411).

### The production of temporary cysts

**Fig 3:**
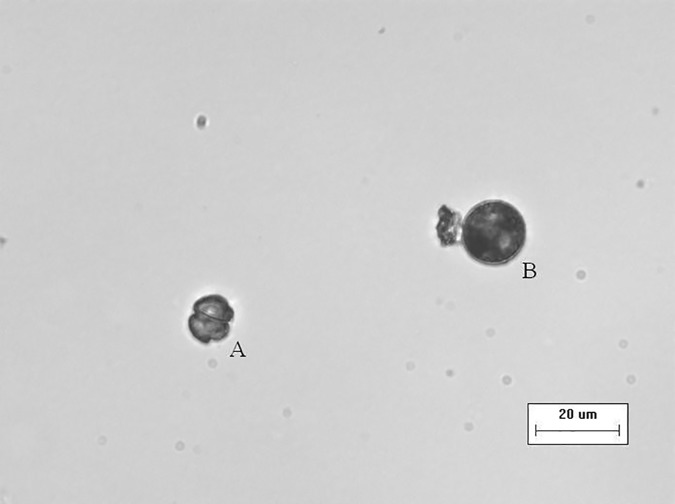
The size and shape of temporary cysts of *K*. *mikimotoi* in the no N treatment on day 8. The photo was taken by the inverted microscope (×40 magnification, IX51 Olympus, Japan).

The formation of temporary cysts were found in the N = 0 treatment beginning on day 8. Fig 3 showed an example of temporary cysts observed under the microscope, the temporary cysts were much bigger than normal cell size and became round in shape (Fig 3B). Although the cellular structure was not indicated by this photo, it was observed that the color of the cellular contents in the temporary cyst was darker than the normal cell and the cell wall became thick, smooth and transparent (Fig 3). The cell volume was calculated considering the cells as round. The average cellular volume of the temporary cysts was 2485.303±541.6348 μm^3^, which is ~ 3.5 times bigger than normal cells (730.0461±305.3773 μm^3^). Cell inhibition rate (PD, %) and temporary cysts production rate (PC, %) on day 8, 11, 14, 17, 20 in the no N treatment were calculated (Table 4). As the culture time increased, the cell inhibition rate increased and cyst production rate decreased (Table 4) resulting in a negative linear relationship (*P*<0.01, *R*^*2*^ = 0.9848).

**Table 4:** The cell inhabition rate (PD, %) and the temporary cysts production rate (PC, %) of *K*. *mikimotoi* in the no N treatment during the culture period.

Time(day)	PD(%)	PC(%)
**8**	80.66	9.12
**11**	89.38	5.19
**14**	89.12	5.44
**17**	93.56	2.68
**20**	92.96	2.74

### Population growth under different N sources

**Fig 4:**
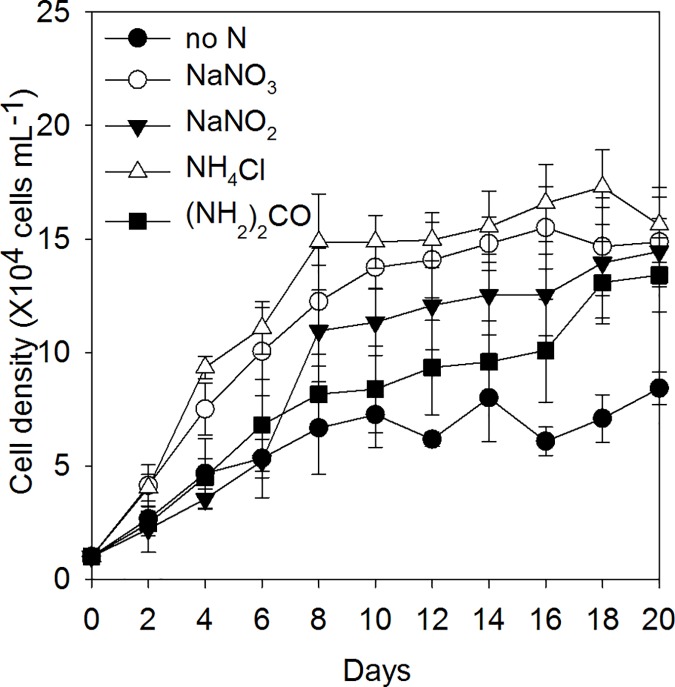
The population growth of *K*. *mikimotoi* under different N sources.

The population growth of *K*. *mikimotoi* under various N sources was shown in Fig 4 and the regression of Logistic model is shown in Table 5. Compared to the no N treatment, *K*. *mikimotoi* cells grew well in the four N forms, *K* values were highest in the NH_4_^+^ treatment (16.2×10^4^ cells mL^-1^) and similar to the other three treatments (13.3–14.9×10^4^ cells mL^-1^) (one-way ANOVA, F = 0.0028, *P* = 0.998). The NH_4_^+^ treatment showed the lowest *T*_*p*_ values and the urea treatment showed the highest *T*_*p*_ values (Table 5). Maximum growth rates (*r*) in the different treatments of N forms were significantly lower than the no N treatment (one-way ANOVA, F = 6.095, *P* = 0.009, Table 5). The urea treatment showed the slowest *r* values and the longest *T*_*p*_ values among the four treatments, indicating a slow growing pattern (Table 5).

**Table 5:** The regression of the logistic model under different nitrogen sources.

Population growth parameters	Growth equation	Carrying capacity *K* (×10^4^ cells mL^-1^)	*r* (d^-1^)	*T*_*p*_ (d)	R^2^
**No N**	*N* = 7.23/(1+e^(2.321–0.727*t*)^)	7.23	0.727	3.2	0.906
**N-NO**_**3**_^**-**^	*N* = 14.89/(1+e^(1.941–0.453*t*)^)	14.89	0.453*	4.3	0.992
**N-NO**_**2**_^**-**^	*N* = 13.93/(1+e^(1.964–0.370*t*)^)	13.93	0.370*	5.3	0.925
**N-NH**_**4**_^**+**^	*N* = 16.21/(1+e^(2.052–0.515*t*)^)	16.21	0.515	4.0	0.978
**N-urea**	*N* = 13.35/(1+e^(1.612–0.216*t*)^)	13.35	0.216*	7.6	0.937

^*^ indicated there was a statically significant difference between this treatment and the corresponding control group (*P*<0.05).

Cells under different N sources were collected in the stationary phase and analyzed for FA composition by GC-MS. The results indicated both the types and relative compositions of fatty acids were variable under different N sources. We found Tetradecanoic acid (14:0), Hexadecanoic acid (16:0), Octadecapentaenoic acid (18:5ω3) and Octadecanoic acid (18:0) in all the treatments, accounting for 8.29%-11.89%, 24.70%-33.32%, 3.62%-16.61% and 4.38%-6.05% (respectively) of the total measured compounds. Except for the four major fatty acids, we also found Docosahexaenoic acid (DHA), Eicosapentaenoic acid (EPA), other unsaturated fatty acids (eg: 16:1ω7c and 18:2ω6), long-chain alkanes or alkenes, sterols and ketones.

The measured compounds were divided into four groups for easy comparison among the different treatments: 1) free fatty acids, 2) long-chain alkanes and alkenes, 3) sterols and ketones, and 4) other compounds (Table 6). Overall, the group of free fatty acids was the dominant compound in all the treatments but the proportion was much less in the NH_4_^+^ treatment (40.61%) compared to the other three treatments (82.13%-84.31%). The free fatty acids include saturated fatty acids (SFA), monounsaturated fatty acids (MUFA) and polyunsaturated fatty acids (PUFA). In the four treatments, SFA accounted for the highest proportion of the total measured compounds, where the proportion was the lowest in the NH_4_^+^ treatment. MUFA was not found in the NH_4_^+^ treatment but was found in the other three treatments ranging from 6.02%-7.29%. The proportion of PUFA in the NH_4_^+^ treatment was ~10 times smaller than the other three treatments, while the proportion of long-chain alkanes or alkenes, sterols or ketones and the other compounds in the NH_4_^+^ treatment were much higher than the other three treatments.

**Table 6:** The classification of organic compounds measured by GC-MS under different nitrogen sources conditions.

Organic compounds		NaNO_3_	NaNO_2_	NH_4_Cl	(NH_2_)_2_CO
**Free fatty acids**	**SFA**	49.43%	44.02%	37.67%	44.31%
	**MUFA**	6.02%	7.11%	0.00%	7.29%
	**PUFA**	26.85%	31.00%	2.94%	32.71%
**Total**		82.30%	82.13%	40.61%	84.31%
**Long-chain alkane or alkene**		14.58%	15.70%	38.72%	13.65%
**Sterols or ketone**		0.91%	0.66%	4.06%	0.73%
**Others**		2.21%	1.51%	16.61%	1.31%

**Fig 5:**
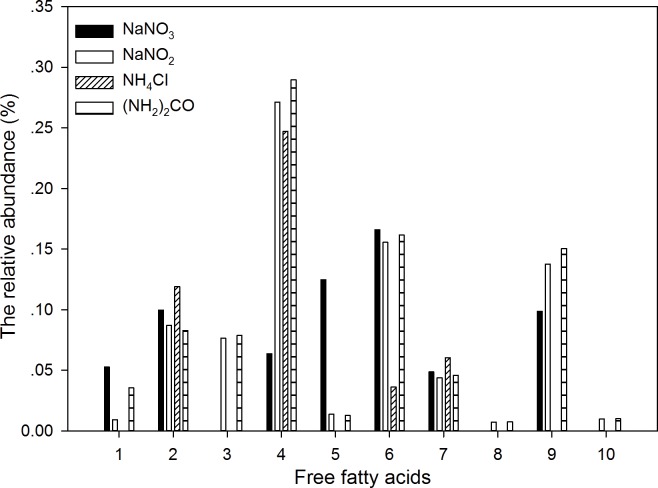
The relative abundance (%) of the major free fatty acids in the total organic compounds measured by GC-MS under different nitrogen sources. The numbers 1–10 indicated 10 kinds of free fatty acids: 1. Dodecanoic acid, 12:0; 2. Tetradecanoic acid, 14:0; 3. 9-Hexadecenoic acid, 16:1ω7c; 4. Hexadecanoic acid 16:0; 5. 9,12-Octadecadienoic acid, 18:2ω6; 6. Octadecapentaenoic acid, 18:5ω; 7. Octadecanoic acid, 18:0; 8. Eicosanoic acid 20:0; 9. 4,7,10,13,16,19-Docosahexaenoic acid 22:6ω3; 10. 5, 8, 11, 14, 17-Eicosapentaenoic acid 20:5ω3.

Fig 5 shows the proportions of 10 major free fatty acids out of the total measured organic compounds. In the 10 kinds of free fatty acids, the abundance of Hexadecanoic acid (16:0), Octadecapentaenoic acid (18:5ω3) and 4, 7, 10, 13, 16, 19-Docosahexaenoic acid (22:6ω3) were the highest among all free fatty acids, and the abundance was lower in the NH_4_^+^ treatment than the other three treatments. All 10 types of free fatty acids were detected in the NO_2_^-^ and urea treatments, while only 5 types of free fatty acids were detected in the NH_4_^+^ treatment. The proportions of Dodecanoic acid and 9, 12-Octadecadienoic acid, 18:2ω6 in the NO_3_^-^ treatment were much higher than the other three treatments. Cells in the NO_2_^-^ and urea treatments had similar proportions for most of the free fatty acids.

## Discussion

*K*. *mikimotoi* blooms often occur in coastal areas where N limitation occurs frequently and N forms are complicated [3, 35–37]. We examined the response of *K*. *mikimotoi* to the variable ambient N conditions, and found: 1) the population growth of *K*. *mikimotoi* was limited by N starvation and recovered quickly after the replenishment of N; 2) temporary cysts were induced by N starvation; 3) *K*. *mikimotoi* grew well in four kinds of N source, but the population growth of *K*. *mikimotoi* varied in different forms of N, as well as the relative compositions of cellular organic compounds.

### The effects of N starvation and resupplement

Under N starvation, we found inhibition of population growth and degradation of cellular chl *a* (Figs 1 and 2). As the initial N concentrations in the media decreased, the growth rates of *K*. *mikimotoi* population started to decrease earlier in the experiment (entered to *T*_*p*_ earlier), and the upper limit of population capacity (*K*) decreased (Table 2). The decline of population growth rates was linked to the degradation of proteins under N deficiency [38]. The loss of cellular chl *a* was also caused by the degradation of proteins (like D1 protein), which contained up to 20% of N [38, 39]. The loss of chl *a* in the cells, resulted in the decline of photosynthetic rates and carbon fixation abilities thus inhibiting energy production and cell division [40]. Under similar N deficient conditions, Zhao et al. [40] did not find apparent population decrease in the diatom *Phaeodactylum tricornutum*, indicating the *K*. *mikimotoi* population was sensitive to changes of ambient N.

After the replenishment of N, cells in all the treatments showed similar recovery growth patterns with cells under normal conditions (similar logistic growth although the treatments with less initial N in the starvation experiment started to recover later.) On day R1, there was no apparent increase of cellular chl *a* content in no N treatment but did increase 2.8 times in N: P = 8:1 treatment, indicating more severe N deficiency in cells leading to slower chl *a* re-synthesis. Different recovery patterns of population growth and cellular chl *a* indicated the recovery of cell division might be independent of re-synthesis of chl *a* at the beginning of the recovery phase. The priority to recover cell divisions over cellular constituents was also found in other species, and this is considered a competitive advantage in the natural environment when nutrient status shifts from deficiency to replete [39, 41, 42]. Although fast recovery is found in both population growth and chl *a* synthesis, neither of them are higher than control values at the end of the recovery experiments, indicating overcompensated growth was not found in our study.

### The production of temporary cysts

Limited by the recording technology in the lab, the transformation of temporary cysts to vegetative cells was not recorded, but the expression of cyst formation related genes are planned to be measured in next step in order to provide more precise evidence for the temporary cysts formations. Although dinoflagellate genomes remain largely unknown, Lin et al. [43] found genes involved in sexual reproduction, cyst formation and germination of dinoflagellate *Symbiodinium kawagutii*, which provided the possibility for us to measure the cyst formation related genes of *K*. *mikimotoi* in the future.Temporary cysts were observed in the no N treatment and there was a negative relationship between the temporary cysts production rate and the cell inhibition rate. The induction of *K*. *mikimotoi* temporary cysts in lab experiments were reported by Uchida et al. [44] and Ma and Pan [45] in which temporary cysts were induced by the allelopathic effect from other microalgal species.Our study indicated N deficiency also could lead to the production of *K*. *mikimotoi* temporary cysts.

In the exponential growth phase (e.g. day 8), the temporary cysts production rate was high while the cell inhibition rate was low, indicating the ability for a fast recovery after the addition of N. On the contrary, there were fewer temporary cysts produced and more cells were inhibited in the stationary phase (e.g. day 20), indicating more a more severe impact to the N deficient cells and less possibility of a fast recovery. Therefore, the process of encystment could be induced by nutrient deficiency but still requires a minimum amount of nutrients. The “threshold effect” of nutrient stress on the encystment of dinoflagellates was also reported by Chen et al. [46] in a study of *Akashiwo sanguinea*. Cysts production rates in our study were between 2.74% and 9.12%, which are within the ranges of encystment rates as reported in previous studies [18, 46].

### The effects of different N sources

Our results showed that *K*. *mikimotoi* cultures grew well when treated with four different forms of N sources, all of which reached cell density observed in *K*. *mikimotoi* blooms in the natural environment (10^5^ cells mL^-1^)[47]. Different phytoplankton groups have different uptake rates and affinities to various forms of N, which are related to the enzyme activities and cellular energy budgets [48, 49]. Due to the influence of human activities, N forms are more variable in coastal areas than in the open ocean [6]. It is possible that the increased proportions of reduced N forms could change the phytoplankton community structure and favor the species with high affinities for reduced N forms [3]. Li et al. [37] reported the *V*_*max-urea*_ of *K*. *mikimotoi* was higher than the HABs species *Prorocentrum donghaiense*. While the *V*_*max-NH4*_ of both species were higher than the diatom species in ECS, thus the increase of reduced ambient N forms would play an important role on triggering the proliferation of HABs of these two species [37].

In our study, *K*. *mikimotoi* population growth showed a slower growth pattern with the addition of urea compared to the other three N sources. The maximum cell densities of *K*. *mikimotoi* population (*K*) did not vary to other three N sources (one-way ANOVA, F = 0.0028, *P* = 0.998), indicating *K*. *mikimotoi* cells could effectively use urea. The utilization of urea in phytoplankton is a process of active uptake, which involves the participation of specific enzymes. These enzymes are not present in all species [50] as shown in Levasseur et al. [51] who reported cellular N deficiency in several species when urea was used as the only N source.

Similar to other dinoflagellate species, *K*. *mikimotoi* population showed the earliest exponential growth and highest carry capacity under NH_4_^+^ among four different N sources [37]. Indicating phytoplankton cells consumed the least energy under NH_4_^+^ compared to NO_3_^-^ and urea, which may lead to the fastest population growth in our results [51]. Phytoplankton can directly utilize NH_4_^+^ to synthesize amino acids, while the use of NO_3_^-^ and NO_2_^-^ requires the participation of nitrate reductase and nitrite reductase, respectively [7]. In the treatments with three inorganic N sources, cells cultured by NO_2_^-^ showed the smallest *K* value and the largest *T*_*p*_ value. Sciandra and Amara [52] pointed out the existence of NO_3_^-^ was necessary to the synthesis of nitrite reductase, therefore the lack of NO_3_^-^ in the NO_2_^-^ treatment may lead to slow population growth in the *K*. *mikimotoi* cultures. The ability to use four different kinds of N provides further evidence that this population has a high competitive ability in changing coastal environments when the proportions of urea and NH_4_^+^ are increasing.

In the treatments of NO_3_^-^, NO_2_^-^ and urea, free fatty acids took account for more than 80% of the total measured organic compounds, when considering the 10 types of major fatty acids measured herein. Cells in the NO_2_^-^ and urea treatments produced more types of fatty acids than the other two treatments, such as 16:1ω7c, 20:0 and 22:6ω3. Free fatty acids in *K*. *mikimotoi* are considered to be sources of toxins, some of which have hemolytic toxicities [53, 54]. Sellem et al. [55] tested the toxic effect of four kinds of PUFA produced by *K*. *mikimotoi*, whose results indicated the toxic effects of 18:4ω and 18:5ω were higher than 20:5ω and 22:6 and they concluded that the toxicities of PUFA were inversely proportional to the length of the carbon chain. The proportion of fatty acids less than 18C (group 1–7) in NO_3_^-^, NO_2_^-^, NH_4_^+^ and urea treatments were 55.57%, 65.71%, 46.26% and 70.73%, respectively, indicating the highest potential toxicities of cells when using urea as N sources. This finding is consistent with former studies: Leong et al. [56] who found higher and different toxin concentrations in urea-grown cells of *Alexandrium tamarense* comparing to NO_3_^—^or NH_4_^+^-grown cells. Shimizu et al. [57] found the toxin concentrations of *Karenia brevis* cells with the addition of urea was 6-fold compared to the addition of NO_3_^-^. Therefore, the increased input of urea in coastal areas might not only result in increasing the frequency of *K*. *mikimotoi* blooms, but also result in the increasing toxicities of *K*. *mikimotoi* cells. On the contrary to culturing with the addition of urea, cells cultured with the addition of NH_4_^+^ had low proportions of fatty acids and high proportions of small molecular organic compounds, indicating potentially low toxicity of cells compared to culturing with the addition of the other three N sources.

## Conclusion

In conclusion, the enhancement of coastal eutrophication changes N dynamics in terms of N: P (N: Si) ratios and its various forms, leading to severe (or alleviated) N limitation and the increased proportions of reduced N and organic N [6, 12]. The formation of temporary cysts under N starvation, fast recovery of population growth after relief of N starvation and the ability to utilize various N sources provide evidence that this strain of *K*. *mikimotoi* could take advantage of changing N conditions in coastal areas. Moreover, we found increased urea proportions in total N concentrations have the potential to lead to slow growing but more toxic *K*. *mikimotoi* cells, while increased NH_4_^+^ might lead to less toxic *K*. *mikimotoi* cells but higher cell densities. All the above results can be taken as evidence of evaluation or applied to models in order to evaluate and predict *K*. *mikimotoi* blooms in dynamical coastal environments. This study was only based on one stain of *K*. *mikimotoi*, whether similar patterns happen to other stains of *K*. *mikimotoi* still needs further investigation.

## Supporting information

S1 File All figures data. (XLSX)
